# Intradiscal treatment of the cartilage endplate for improving solute transport and disc nutrition

**DOI:** 10.3389/fbioe.2023.1111356

**Published:** 2023-02-27

**Authors:** Mohamed Habib, Shayan Hussien, Oju Jeon, Jeffrey C. Lotz, Peter I-Kung Wu, Eben Alsberg, Aaron J. Fields

**Affiliations:** ^1^ Department of Orthopaedic Surgery, University of California, San Francisco, San Francisco, CA, United States; ^2^ Department of Mechanical Engineering, Al Azhar University, Cairo, Egypt; ^3^ Department of Biomedical Engineering, University of Illinois, Chicago, IL, United States

**Keywords:** cartilage endplate, intervertebral disc degeneration, biotransport, spine biomechanics, low back pain, tissue engineering

## Abstract

Poor nutrient transport through the cartilage endplate (CEP) is a key factor in the etiology of intervertebral disc degeneration and may hinder the efficacy of biologic strategies for disc regeneration. Yet, there are currently no treatments for improving nutrient transport through the CEP. In this study we tested whether intradiscal delivery of a matrix-modifying enzyme to the CEP improves solute transport into whole human and bovine discs. Ten human lumbar motion segments harvested from five fresh cadaveric spines (38–66 years old) and nine bovine coccygeal motion segments harvested from three adult steers were treated intradiscally either with collagenase enzyme or control buffer that was loaded in alginate carrier. Motion segments were then incubated for 18 h at 37 °C, the bony endplates removed, and the isolated discs were compressed under static (0.2 MPa) and cyclic (0.4–0.8 MPa, 0.2 Hz) loads while submerged in fluorescein tracer solution (376 Da; 0.1 mg/ml). Fluorescein concentrations from site-matched nucleus pulposus (NP) samples were compared between discs. CEP samples from each disc were digested and assayed for sulfated glycosaminoglycan (sGAG) and collagen contents. Results showed that enzymatic treatment of the CEP dramatically enhanced small solute transport into the disc. Discs with enzyme-treated CEPs had up to 10.8-fold (human) and 14.0-fold (bovine) higher fluorescein concentration in the NP compared to site-matched locations in discs with buffer-treated CEPs (*p* < 0.0001). Increases in solute transport were consistent with the effects of enzymatic treatment on CEP composition, which included reductions in sGAG content of 33.5% (human) and 40% (bovine). Whole disc biomechanical behavior—namely, creep strain and disc modulus—was similar between discs with enzyme- and buffer-treated CEPs. Taken together, these findings demonstrate the potential for matrix modification of the CEP to improve the transport of small solutes into whole intact discs.

## 1 Introduction

Back pain is the leading cause of disability and is a major public health problem worldwide (Haralson & Zuckerman, 2009; Vassilaki & Hurwitz Dc, 2014; Matta et al., 2017; Fernandez-Moure et al., 2018). In certain subgroups of back pain patients, intervertebral disc degeneration may cause pain (Chou et al., 2011). Current medical interventions for disc degeneration are surgical in nature and are often unsuccessful, which motivates development of non-operative alternatives. Non-operative approaches to regenerate the disc and alleviate pain include delivering genes (Woods et al., 2011), growth factors (Bae & Masuda, 2011; Ju et al., 2022), cells (Velikonja et al., 2014; Ju et al., 2022), or other small molecules (Gawri et al., 2013; Wang et al., 2021) to promote cell proliferation, stimulate anabolic activities, and reduce catabolism and inflammation. Importantly, these approaches all require a rich nutrient supply to sustain higher cell numbers or metabolic rates (Horner & Urban, 2001; Fernandez-Moure et al., 2018). However, disc degeneration in humans is typically accompanied by a restricted nutrient supply (Fields et al., 2018; Huang et al., 2014; Urban et al., 2004), which may limit the clinical utility and effectiveness of these therapies (Huang et al., 2014; Zhu et al., 2016; Binch et al., 2021; Ju et al., 2022). Yet, there are no existing treatments to improve disc nutrient supply. Developing treatment strategies to improve disc nutrition may therefore aid in slowing or reversing degeneration, and could enhance the regenerative potential of biologic therapies that increase nutrient demands inside the disc.

Proper disc nutrition involves nutrient and metabolite exchange between the nucleus pulposus (NP) cells and vertebral capillaries (Maroudas et al., 1975; Nachemson et al., 1970; Ohshima et al., 1989; Urban et al., 1977), and several factors can impair the normal patterns of solute exchange. For example, nutrients entering the disc and exiting metabolites must pass through the cartilage endplates (CEPs), and solute passage could be slowed or blocked by changes to the composition of the CEP matrix, including dehydration (Antoniou et al., 1996), mineralization (Wong et al., 2019; Benneker et al., 2005; Grant et al., 2016) and fibrosis (Antoniou et al., 1996; Wong et al., 2019). For example, we found that CEPs with higher amounts of collagen, sulfated glycosaminoglycan (sGAG), and mineral hindered nutrient diffusion, thereby impairing NP cell survival and function inside diffusion chambers (Wong et al., 2019). Moreover, those same deficits in CEP composition were also associated with significantly worse disc degeneration in low back pain patients (Bonnheim et al., 2022).

Motivated by those findings, we explored a matrix modification strategy for improving disc nutrition that involves enzymatically reducing the amounts of collagen and aggrecan in the impermeable and fibrotic CEPs (Dolor et al., 2019). Treatment of human cadaveric CEPs with recombinant human MMP-8 enzyme, which is one of the matrix metalloproteinases that has a high specificity for type II collagen and aggrecan, reduced sGAG content in a dose-dependent manner. Importantly, reductions in CEP matrix content with MMP-8 treatment improved the NP cell viability inside diffusion chambers, indicating improved CEP permeability to nutrients (Dolor et al., 2019). Although the diffusion chambers represent a useful model system for studying the effects of CEP permeability enhancement, the chambers do not mimic the complex microenvironment or loading of an intact intervertebral disc, and thus, the effects of CEP permeability enhancement on solute transport into whole, intact discs remain unclear. The objectives of this study were to: 1) develop a model for the controlled, intradiscal delivery of a matrix-modifying enzyme to the CEP; and 2) measure solute transport into whole human and bovine discs following intradiscal treatment of the CEP with a matrix-modifying enzyme.

## 2 Methods

### 2.1 Bovine tissues

Nine intact bovine coccygeal motion segments were harvested from three oxtails (3 segments per oxtail) acquired within 2 h of slaughter from a local abattoir (approximate age range of the steers: 18–22 months old). After harvesting the motion segments, a 17G Crawford needle with a plasma-mediated coblation wand (ArthroCare 2000) was inserted through the posteriolateral annulus directed toward one CEP in each disc, the wand was energized on a low-power setting (2 W) for 10 s as the needle was advanced to the distal annulus, and the coblated tissue was debrided with PBS. This coblation step was necessary for overcoming the high back pressure in the otherwise healthy bovine discs. Following coblation, the wand was removed with the needle in place, and the motion segments were randomly assigned to three groups (n = 3 motion segments/group; Table 1): collagenase—0.15 U in 50 µl of slow-release alginate carrier; control buffer–HBSS in 50 µl of alginate carrier; or sham–needle insertion only.

**TABLE 1:** Bovine coccygeal (CC) disc allocation to study groups.

Steer #	Sham	Control buffer	Collagenase
**Bovine 1**	CC3	CC1	CC2
**Bovine 2**	CC1	CC2	CC3
**Bovine 3**	CC2	CC3	CC1

### 2.2 Human tissues

Five human cadaveric lumbar spines (age range: 38–66 years old; mean age: 56 ± 10 years) were obtained from donors with no medical history of musculoskeletal disorders (UCSF Willed Body Program). From these spines, ten intact motion segments (2 motion segments per spine) were harvested and randomly assigned to two groups (n = 5 motion segments/group; Table 2); collagenase – 0.15 U in 50 µl of alginate carrier; or control buffer–HBSS in 50 µL of alginate carrier). Prior to spine harvest all cadavers underwent MR imaging (GE Discovery MR 750 W 3T; GE Healthcare). Imaging consisted of a sagittal fast spin-echo T2-weighted sequence and a 3D multi-echo UTE cones mapping sequence. Sequence parameters were identical to those reported previously (Wang et al., 2021). To ensure that both motion segments from the same donor were structurally intact and of a similar degree of degeneration, we selected motion segments from levels with intact CEPs and similar Pfirrmann degeneration grades (Supplementary Materials).

**TABLE 2:** Human cadaver demographic data and lumbar disc allocation to study groups.

				Control buffer		Collagenase	
Donor #	Age (yrs)	Sex (F/M)	BMI (kg/m^2)	level	Pfirrmann Grade	level	Pfirrmann Grade
Donor 1	56	F	18.3	L2-L3	III	L3-L4	III
Donor 2	57	F	24.8	L4-L5	III	L3-L4	III
Donor 3	25	M	23.7	L3-L4	II	L4-L5	II
Donor 4	61	M	27.4	L3-L4	III	L4-L5	III
Donor 5	63	F	21.1	L4-L5	III	L3-L4	III

### 2.3 Alginate preparation

For alginate purification, sodium alginate (10 g, Protanal LF 20/40) was dissolved in 1,000 ml ultrapure deionized water (diH_2_O), dialyzed against diH_2_O (MWCO 3500; Spectrum Laboratories Inc.) for 3 days, treated with activated charcoal (0.5 mg/100mL, 50–200 mesh, Fisher) for 30 min, filtered (0.22 μm filter) and lyophilized.

Oxidized alginates (OA) were prepared by reacting sodium alginate (Protanal LF 20/40, 196,000 g/mol, FMC Biopolymer) with sodium periodate (Sigma) using a previously described method (Jeon et al., 2017). Briefly, sodium alginate (10 g) was dissolved in ultrapure deionized water (diH_2_O, 900 ml) overnight. Sodium periodate (543 and 1,085 mg) was dissolved in 100 ml diH_2_O and added to separate alginate solutions to achieve different degrees of theoretical alginate oxidation (5% and 10%, respectively) under stirring in the dark at room temperature for 24 h. The OAs were purified by dialysis against diH_2_O (MWCO 3500; Spectrum Laboratories Inc.) for 3 days, treated with activated charcoal (0.5 mg/100ml, 50–200 mesh, Fisher) for 30 min, filtered (0.22 μm filter) and lyophilized. The actual oxidation (4.2% ± 0.5% and 8.5% ± 0.3%) was determined by using the AmpliteTM Colorimetric Aldehyde Quantitation Kit (AAT Bioquest Inc.) according to the manufacturer’s instructions.

We compared the release kinetics of the different alginate formulations (0% oxidation, 5% oxidation, 10% oxidation) in an *ex-situ* pilot study (Supplementary Materials), and based on the observed release kinetics, we used the 0% oxidation alginate for all intradiscal injections.

### 2.4 Intradiscal CEP treatment

**FIGURE 1:**
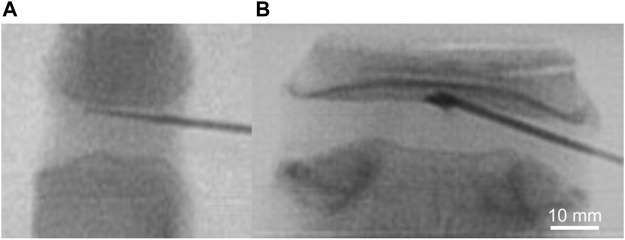
Intradiscal delivery of collagenolytic enzyme treatment *via* 17G Tuohy needle to superior CEP of **(A)** bovine coccygeal and **(B)** human lumbar intervertebral discs under fluoroscopic guidance.

Enzymatic treatment or control buffer was delivered to the CEP under fluoroscopic guidance. Briefly, a 17G Tuohy needle (Medline, item: PAIN8001) was inserted through the posterolateral annulus fibrosus and advanced to the central region of the superior CEP in each disc (Figure 1). After needle placement, 50 µL of either collagenase or control buffer that was suspended in the alginate carrier was injected to the CEP, and then fibrin sealant (TISSEEL, Baxter International Inc., CA, United States) was applied to the annulus during needle retraction. All motion segments were then incubated in protease inhibitor cocktail dissolved in PBS buffer according to the manufacturer’s protocol (Sigma-Aldrich, catalog number P2714) for 18 h at 37 °C.

### 2.5 Transport experiment

**FIGURE 2:**
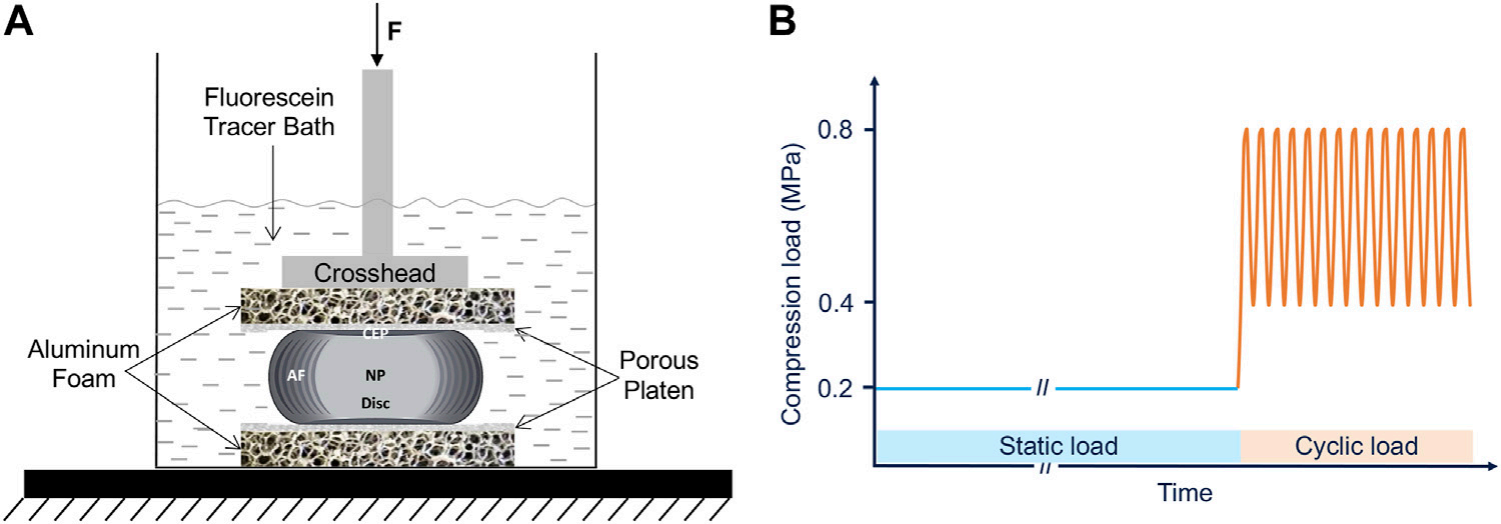
Experimental setup **(A)** and the compression testing protocol **(B)** of the whole intact disc while being immersed in fluorescein tracer solution.

**FIGURE 3:**
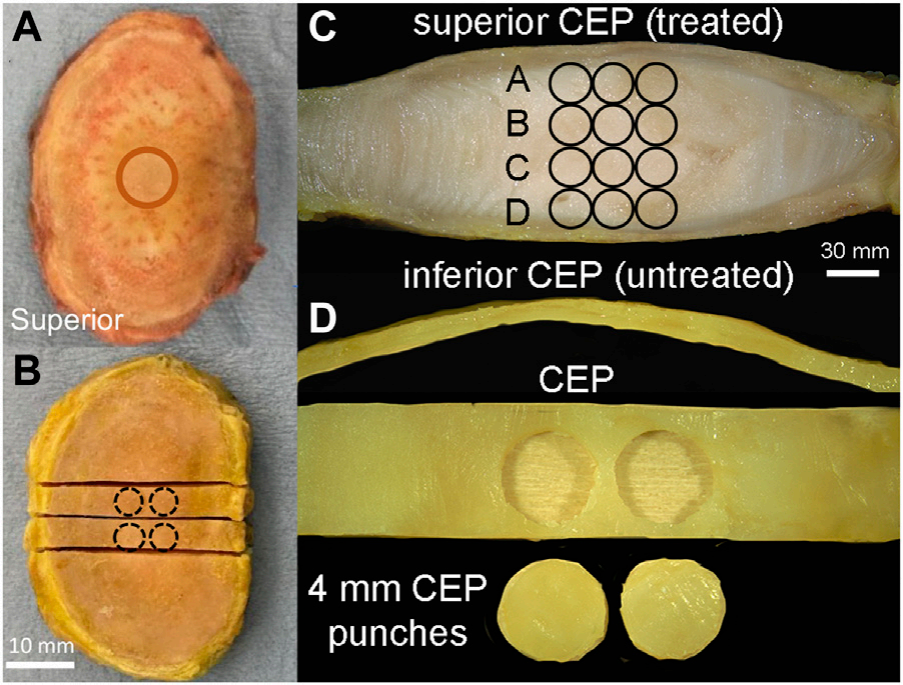
**(A)** Prior to compression testing, whole discs were isolated from the surrounding soft tissue, and the bony endplate was removed with a burring tool. Circle indicates alginate gel placement underneath the superior CEP as in Figure 1B. **(B)** After compression testing, discs were snap-frozen in liquid nitrogen and two 5 mm-wide mid-sagittal slabs were removed for further biochemical and fluorescein measurement. Circles indicate the locations of CEP punches harvested for biochemical measurements. **(C)** Mid-sagittal slab showing NP tissue sample positions (three 2 mm punches per inferior-superior positions) relative to treated and un-treated CEPs. **(D)** Two 4 mm-diameter punches were harvested from the central region of each CEP in the mid-sagittal slab **(B)**.

A custom setup was used to evaluate the effects of CEP permeability enhancement on solute transport into the disc (Figure 2). To eliminate any potentially confounding effects of differences in bony endplate structure within and between species, the inferior and superior bony endplates were carefully removed from the motion segments with a burring tool (Figure 3A). Several x-ray images (Faxitron Cabinet X-ray Systems model 43855A) were taken during the bone removal process to ensure the complete removal of all bone tissues and to calculate the cross-sectional area of the isolated discs (ImageJ, NIH).

The isolated discs were then sandwiched between porous stainless-steel platens (0.8 mm hole diameter, 1.8 mm thickness and 17.9% hole coverage) and open-cell porous aluminum foam blocks with similar porosity and stiffness of vertebral trabecular bone (Duocel^®^ aluminum foam disc, 7%–9% relative density, ERG Aerospace Corp, Oakland, CA) while submerged in 200 mL of fluorescein tracer solution (376 Da; 0.1 mg/mL in PBS) (Figure 2). A servo-hydraulic load frame (MTS Bionix 858; MTS Systems Corp., Eden Prairie, MN) was used to apply loads resulting in physiologic disc pressures. Specifically, we applied 6 h of static compression loads that represent the lower end of intradiscal pressure range measured during various static postures (0.2 MPa) (Nachemson, 1966; Wilke, et al., 1999), followed by 1.6 h of cyclic compressive loads resulting in a pressure range (0.4–0.8 MPa) and frequency (0.2 Hz) that is similar to those measured in the disc during walking (Wilke, et al., 1999).

### 2.6 NP and CEP tissue sampling

Immediately after the transport experiments, discs were snap frozen in liquid nitrogen to prevent any further fluorescein dispersion. Next, two 5-mm-thick-coronal slabs were cut with a low-speed diamond saw (IsoMet, Buehler, IL) (Figure 3B). From each slab, three NP samples were collected using a 2-mm biopsy punch at four superior-inferior locations (Figure 3C). The superior and inferior CEP layers were then carefully removed from the discs and trimmed of any residual NP tissue (Figure 3D). Two CEP samples were collected from each intact CEP using a 4-mm biopsy punch (Figure 3D). All NP and CEP samples were massed on microbalance, dried by lyophilization, and completely digested in papain for subsequent assays.

### 2.7 Fluorescein concentration

NP tissue digests were assayed for fluorescence on a SpectraMax iD5 microplate reader (Molecular Devices, San Jose, CA), and the fluorescence readings (ex. 485 nm, em. 525 nm) referenced to a standard curve of known fluorescein concentrations. Fluorescein concentrations inside the NP samples were calculated based on the measured water content from lyophilization and were compared between site-matched locations in the discs having collagenase-treated vs buffer-treated CEPs.

### 2.8 CEP biochemical composition

CEP tissue digests were assayed for sGAG content using a dimethylmethylene blue assay (DMMB) (Farndale et al., 1986; Sampson et al., 2019). The digest absorbance was measured with a microplate reader at 595 nm and referenced to a standard curve of known chondroitin sulfate concentrations.

Aliquots of CEP digests were also hydrolyzed in 6 N HCL for 18 h at 110°C. Neutralized acid hydrolysates were then assayed for total collagen content using a chloramine-T colorimetric assay (Fields et al., 2014), referencing the absorbance measurements to a standard curve of known hydroxyproline concentrations.

The total sGAG and collagen contents in the CEP tissues were used to evaluate the effect of the enzymatic treatment on the CEP matrix composition.

### 2.9 Whole disc biomechanical properties

The axial force, crosshead displacement and time data were collected during the transport experiment and processed with a custom script (MATLAB, Mathworks; Natwick, MA) that calculates the disc modulus and the total creep strain. Disc modulus was considered as the slope of the stress-strain curve at maximum value of the stress in the linear part of the static compression phase. The total creep strain was considered as the ratio between total displacement at the end of the static compression phase and the initial height of the disc.

### 2.10 Statistics

Mixed effects models accounting for multiple measurements per subject *via* random intercept were used to estimate the least-squares mean fluorescein concentration at each NP location and the least-squares mean sGAG and collagen content in the CEP, and Tukey HSD *post hoc* tests were used to test for differences between groups. One-way ANOVA was used to compare the whole-disc compressive creep strain and modulus between groups. Statistical analyses were performed in JMP Pro 15; *p* < 0.05 was considered statistically significant.

## 3 Results

### 3.1 Effects of enzymatic treatment on solute transport

**FIGURE 4:**
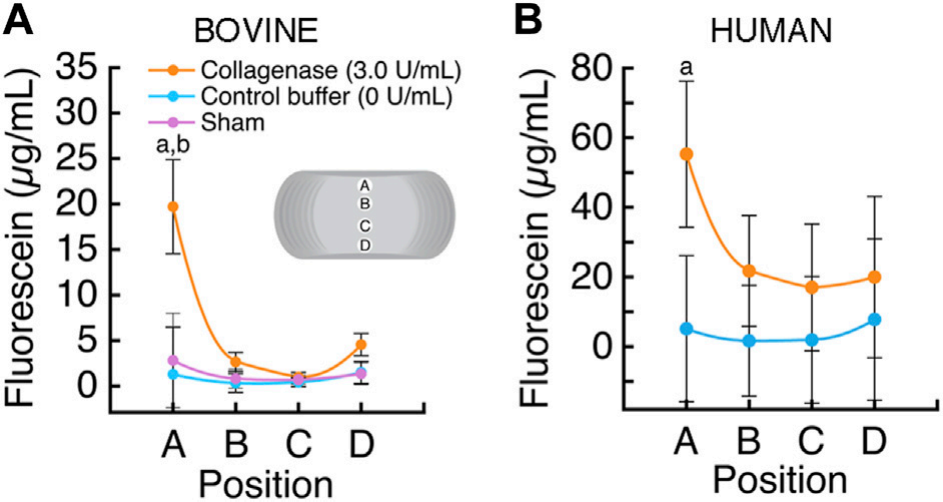
**(A)** The average fluorescein concentration (mean ± SD; n = 3 discs/group) was significantly higher in the bovine NP proximal to the collagenase-treated superior CEP, position A (a *p* < 0.0001 vs control buffer; b *p* < 0.0001 vs sham; *p* > 0.10 all other comparisons). **(B)** The average fluorescein concentration (mean ± SD; *n* = 5 discs/group) was significantly higher in the human NP proximal to the collagenase-treated superior CEP, position A (a *p* < 0.0001).

Intradiscal enzymatic treatment of the CEP significantly improved solute transport into the bovine coccygeal discs (Figure 4A). Specifically, in the NP near to the superior treated CEP, fluorescein tracer concentration was 14-fold higher in the discs that had CEPs treated with collagenase than in the discs that had CEPs treated with control buffer (19.72 ± 5.17 μg/mL vs 1.41 ± 1.11 μg/mL; *p* < 0.0001) and 7-fold higher than discs with untreated CEPs (2.81 ± 1.79 μg/mL; sham). In discs with buffer-treated and sham CEPs, fluorescein concentrations were generally highest near the CEPs, at positions A (buffer: 1.41 ± 1.11; sham: 2.81 ± 1.79 μg/mL) and D (buffer: 1.48 ± 1.66; sham: 1.35 ± 0.52 μg/ml), and lowest in the center of the discs, at positions B (buffer: 0.33 ± 0.14; sham: 0.81 ± 0.34 μg/ml) and C (buffer: 0.43 ± 0.27; sham: 0.71 ± 0.25 μg/ml).

Similarly, intradiscal enzymatic treatment of the CEP improved solute transport into human lumbar discs (Figure 4B). The fluorescein concentration in the NP near to the superior treated CEP, at position A, was 10.8-fold higher inside the discs that had CEPs treated with collagenase than in the discs that had CEPs treated with the control buffer (55.27 ± 31.11 μg/mL vs 5.14 ± 3.54 μg/mL; *p* < 0.0001). In the discs with buffer-treated CEPs, fluorescein concentration varied with position; concentrations were generally highest adjacent to the CEPs, positions A (5.14 ± 3.54 μg/ml) and D (7.14 ± 2.26 μg/ml), and lowest in the center of the discs, at positions B (0.58 ± 0.18 μg/ml) and C (0.62 ± 0.19 μg/ml).

### 3.2 Effects of enzymatic treatment on CEP matrix composition

**FIGURE 5:**
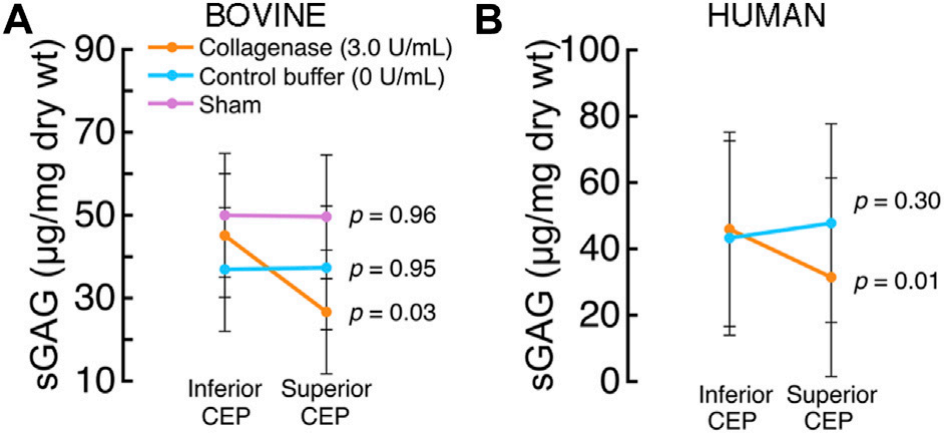
**(A)** In the bovine discs that underwent collagenase treatment, sGAG content in the treated (superior) CEP was significantly lower than the sGAG content in the untreated (inferior) CEPs (*p* = 0.03, inferior *versus* superior paired *t*-test; mean ± SD; n = 3 discs/group) **(B)** In the human discs that underwent collagenase treatment, sGAG content in the treated (superior) CEPs was significantly lower than the sGAG content in the untreated (inferior) CEPs (*p* < 0.01, inferior *versus* superior paired *t*-test; mean ± SD; n = 5 discs/group).

Intradiscal enzymatic treatment significantly reduced the amount of solute-blocking matrix constituents in both bovine and human CEPs. Specifically, collagenase treatment reduced sGAG content, with treated bovine CEPs showing, on average, 40% lower sGAG content than their paired untreated CEPs (*p* = 0.03; Figure 5A). The CEPs that were untreated as well as those that were treated with the control buffer had similar sGAG contents. Likewise, the same enzyme dose delivered intradiscally to human CEPs significantly reduced sGAG content, with the superior CEPs showing 33.5% lower sGAG content, on average, than their paired inferior CEPs (*p* = 0.01; Figure 5B).

**FIGURE 6:**
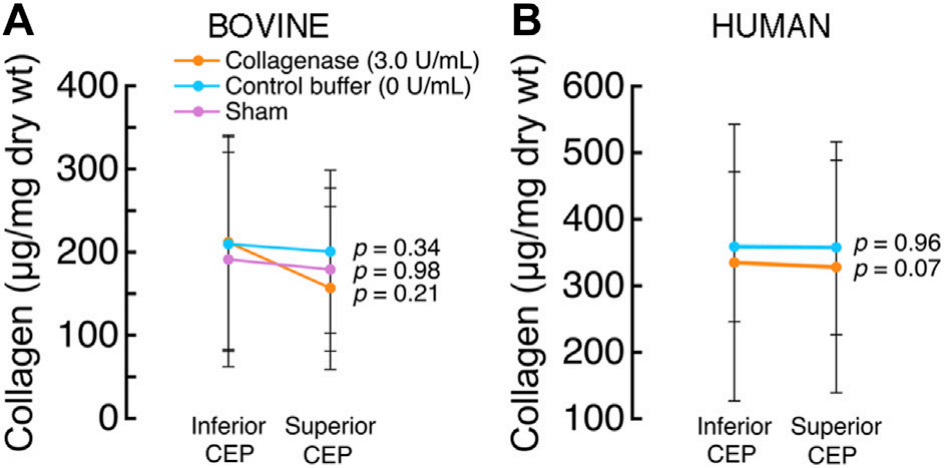
**(A)** In the bovine discs that underwent collagenase treatment, collagen content in the treated (superior) CEPs was similar to that of the untreated (inferior) CEPs (*p* = 0.21, paired *t*-test; mean ± SD; n = 3 discs/group) **(B)** In the human discs that underwent collagenase treatment, collagen content in the treated (superior) CEPs was similar to that in the untreated (inferior) CEPs (*p* = 0.07, paired *t*-test; mean ± SD; n = 5 discs/group).

Collagenase treatment tended to associate with lower collagen content in the treated CEPs, but the differences were not statistically significant. Specifically, mean collagen content was lower in the superior treated CEPs (156.79 ± 84.55 μg/mg dry wt) than their paired inferior CEPs (233.39 ± 85.73 μg/mg dry wt; *p* = 0.21; Figure 6A), but the difference did not reach statistical significance. In the human discs that underwent collagenase treatment, collagen content in the treated superior CEPs (327.75 ± 188.65 μg/mg dry wt) tended to be lower than their paired untreated CEPs (334.7186 ± 208.01 μg/mg dry wt; *p* = 0.07; Figure 6B).

### 3.3 Effects of enzymatic treatment on NP tissue composition

To understand how the enzymatic treatment delivered to the CEP affected the composition of the NP tissue, we measured the sGAG content in the human NP tissue samples that were assayed for fluorescein (Table 3). Collagenase treatment had a small, localized effect on the sGAG content of the adjacent NP tissue. Specifically, mean sGAG of NP samples adjacent to the collagenase-treated CEP at position A was 15.2% lower than the NP tissue samples adjacent to the untreated CEP at position D in the same discs (*p* = 0.027) and 13.8% lower than the tissue NP samples at position B in the same discs (*p* = 0.026). Comparisons of mean sGAG between the NP tissue samples at the other positions were not significant.

**TABLE 3:** Effects of intradiscal treatment on sGAG content (µg/mg dry) at the NP different positions.

Treatment	Position A	Position B	Position C	Position D
**Control**	231.9 ± 107.4	222.9 ± 133.2	225.3 ± 107.6	223.1 ± 95.4
**Collagenase**	207.9 ± 133.8	241.3 ± 132.9^a^	218.8 ± 105.4	245.2 ± 98.2^a^

^a^ *p* < 0.05 vs Position A by paired *t*-test. All other comparisons *p* > 0.05.

### 3.4 Effects of enzymatic treatment on whole-disc biomechanical behavior

**FIGURE 7:**
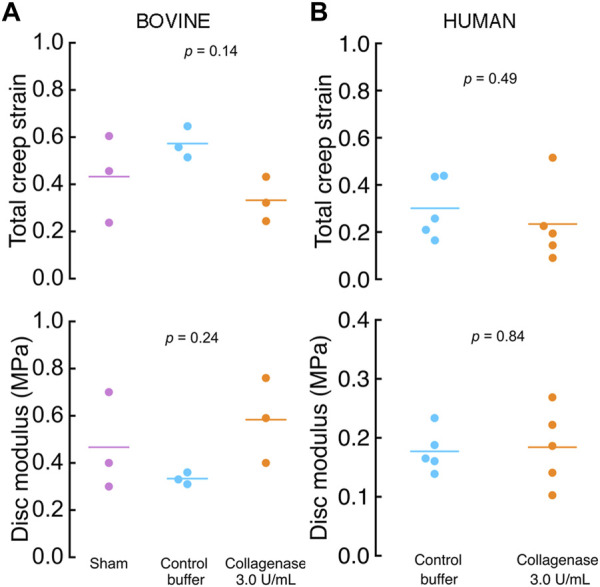
Whole-disc compressive creep strain (top) and modulus (bottom was similar in **(A)** bovine and **(B)** human discs with collagenase-treated CEPs vs control CEPs. *p-*values are from one-way ANOVA.

To determine the biomechanical effects of CEP matrix modification at the whole-disc level, we compared the compressive modulus and total creep strain between groups by ANOVA. In the bovine discs, total creep strain and compressive modulus were similar between discs with the collagenase-treated CEPs, buffer-treated CEPs, and untreated CEPs (Figure 7A). Likewise, in the human discs, total creep strain and compressive modulus were similar between discs with collagenase-treated and buffer-treated CEPs (Figure 7B).

## 4 Discussion

Here we investigated intradiscal treatment to modify the matrix of the CEP to enhance the transport of small solutes into whole intervertebral discs. Collagenase enzyme was loaded into a degradable hydrogel carrier and then delivered *via* needle to the superior CEP under fluoroscopic guidance. Results show that collagenase treatment (0.15 U) significantly reduced the amount of sGAG in the treated CEP: enzymatic treatment of human lumbar and bovine coccygeal CEPs reduced sGAG by 33.5% and 40%, respectively (Figure 5). Enzymatic treatment also led to reductions in collagen content, but the treatment effects were heterogeneous, and the differences in CEP collagen content between treated and untreated CEPs of the same disc were not statistically significant. Importantly, enzymatic treatment of the CEP coincided with greater transport of 376-Da fluorescein into the nucleus pulposus under static and cyclic compressive loads. Specifically, NP tissue adjacent to collagenase-treated human and bovine CEPs had 10.8-fold (human) and 14-fold (bovine) higher fluorescein concentrations compared to the NP tissue adjacent to buffer-treated CEPs after loading (Figure 4). The similar pattern of the findings in bovine discs with relatively healthy CEP and NP tissues and human discs with more degenerated CEP and NP tissue lends confidence in the generalizability of the treatment mechanism and the overall magnitude of the treatment effect. In our previous study of human CEPs, we observed that *ex situ* treatment of CEPs with truncated human recombinant MMP-8 reduced sGAG content in a dose-dependent manner, increased fluorescein uptake into unloaded CEPs, and enhanced nutrient transport and NP cell viability (Dolor et al., 2019). Our new findings demonstrate for the first time the concept of CEP matrix modification to whole, compressively loaded discs, and taken together, these findings support the potential for matrix modification of the CEP to improve the transport of small solutes in the size range of essential nutrients, e.g., glucose (180 Da).

The goal of CEP matrix modification is to reduce solute-blocking matrix constituents, improve the CEP’s permeability to nutrients, and enhance disc cell viability/function (Dolor et al., 2019; Wong et al., 2019). In our previous study, reducing the amount of sGAG in the CEP by 6%–22% coincided with increases in passive fluorescein uptake by 16%–24% (Dolor et al., 2019). *In vivo*, the disc is subjected to a combination of static and cyclic loads, which improves solute transport through the CEP by inducing convection (Sampson et al., 2019). The effects of fluid flow on solute convection may explain why a similar enzyme dose used in the current study produced a larger increase in fluorescein transport through the CEP than observed with passive diffusion.

The CEP is rich in type II collagen (Schollmeier et al., 1976; Roberts et al., 1991; Moore, 2006; Nosikova et al., 2012) and aggrecan. We used a commercial form of collagenase *p* from *Clostridium* histolyticum (C. histolyticum) for matrix modification, as this enzyme can break down the peptide bonds in four types of collagens (I, II, III, and IV) (Alipour et al., 2016) and has proteolytic activity on proteoglycans. Matrix metalloproteinases (MMPs) also have a high specificity for type II collagen and aggrecan (Nagase and Kashiwag, 2003; Dolor et al., 2019). In the current study, we treated both human and bovine CEPs, so we chose a generic collagenase enzyme rather than a specific human recombinant MMP. Nevertheless, the effects of collagenase *p* on human CEP composition appeared similar to those observed with truncated human MMP-8 (Dolor et al., 2019), and together our findings suggest that both enzymes reduce sGAG content in the CEP and have more subtle effects on the collagen. Although collagen content in the collagenase-treated CEPs was typically lower than buffer-treated CEPs, these differences were not statistically significant (Figure 6). One possible explanation is that cleaved collagen fragments remained intertwined in the CEP matrix, and greater exposure times are required for the removal of these fragments (Jiang et al., 2020).

Targeted delivery of matrix-modifying enzymes to the CEP presents several technical challenges. We chose a 50 µl volume of injectate based on preliminary intradiscal pressure testing; injecting larger volumes increased the back pressure and made it difficult to prevent leakage. Choice of the hydrogel carrier was made to control the release of the enzymatic treatment (Supplementary Materials), and intradiscal delivery was performed using a 17G Tuohy epidural needle with a curved end at the needle’s tip that allows the hydrogel to exit laterally at a 45-degree angle. Combined with the oblique trajectory of the needle, it was possible to deliver the treatment to the center of the desired CEP (Figure 1). We treated one CEP from each disc to demonstrate proof-of-principle and to ensure the non-treated CEP would act as an intra-disc control. At this point, it is not clear if treating one CEP is sufficient to improve disc nutrition or if treating both CEPs is necessary. More extensive matrix modification of one or both CEPs could be achieved by redirecting the needle to reach different areas of the target CEP; however, redirecting the needle within the disc to treat multiple locations is challenging and may require more specialized instrumentation. A related challenge is localizing the enzyme and minimizing the effects on the nucleus pulposus tissue. For example, we found that NP tissues adjacent to these enzyme-treated CEPs had 15% lower sGAG content compared to the NP tissues adjacent to the untreated CEP of the same disc (Table 3). This suggests that other strategies may be needed to further reduce off-target treatment effects. For example, attaching the enzyme to large liposomal nanoparticles may limit enzyme mobility and help reduce unwanted enzyme migration in the NP (Dolor et al., 2019).

Proper CEP function requires balancing conflicting biotransport and biomechanical demands, and thus, successful matrix modification strategies for improving biotransport must be sensitive to any biomechanical effects. Specifically, excessive cleavage and removal of collagen fibrils or proteoglycans in the CEP could inadvertently lower disc modulus and risk clinically significant disc injury. The collagen network helps resist transverse CEP stretching that arises during disc compression (Fields et al., 2010; Fields et al., 2014; DeLucca et al., 2016), and proteoglycans in the CEP are believed to help prevent the loss of proteoglycan aggregates from the nucleus pulposus (Roberts et al., 1996). We found that discs with enzyme-treated CEPs had similar whole-disc modulus and total creep strain as buffer-treated discs (Figure 7), which suggests that the modest dose used here was sufficient to improve solute transport without impacting whole-disc behavior under static and cyclic compressive loading. Additional mechanical testing with more complex loading protocols is needed to further understand the biomechanical effects of CEP matrix modification.

This study had several limitations. First, we removed the bony endplates before the transport experiments in order to isolate the effects of CEP matrix composition on solute transport into the discs and to facilitate comparison between species. For example, bovine spinal units have vertebral epiphyses and growth plates that persist through adulthood, whereas adult human discs do not. Also, the bony endplate is much thicker in bovine coccygeal vertebrae than human lumber vertebrae. Given the total permeability of the bony and cartilaginous endplates is more strongly correlated with CEP permeability than with bony endplate permeability (Rodriguez et al., 2011), removing the bony endplates is unlikely to have affected our conclusions about the relative increase in solute transport into the disc with enzymatic treatment. Further, the bony endplate removal was verified by imaging to not disrupt the CEP. A second limitation is that we used one solute size in the transport experiments, and permeability enhancement may be size-dependent. We focused on 376 Da-sized tracer because of its similarity to glucose (180 Da), which is vital to NP cell survival. The effects of enzymatic treatments on large solute filtration by the CEP are also important and remain unknown. A third limitation is that we did not measure localized mechanical properties of the CEP tissue, which are also physiologically important (Lotz et al., 2013; Fields et al., 2014; Fields et al., 2018). Finally, we did not assess the biologic effects of matrix modification in this cadaveric study. To address this, ongoing work is using cell and organ culture models to assess treatment safety and efficacy, including characterizing the cellular response to the by-products from CEP matrix proteolysis.

## 5 Conclusion

We found that intradiscal delivery of a matrix-modifying enzyme to the CEP significantly reduced the amount of sGAG. Treating the CEP in this manner dramatically enhanced small-solute transport into the disc. This is an important step towards *in vivo* pre-clinical studies. Future studies should focus on optimizing enzyme delivery and release, as well as on discovering the native cells’ response to matrix-modifying enzymes and the by-products from matrix proteolysis.

### 5.1 Significance

Strategies for enhancing nutrient transport across the CEP may have the potential to slow or reverse disc degeneration. Our new results are significant because they suggest that intradiscal delivery of a matrix-modifying enzyme to the CEP can effectively reduce solute-blocking matrix constituents and thereby improve solute transport. This treatment strategy could stand alone or possibly be combined with biologic regenerative therapies that target the NP and increase nutrient demands.

## Data Availability

The original contributions presented in the study are included in the article/Supplementary Material, further inquiries can be directed to the corresponding author.
