# Supplementary material for: Intradiscal treatment of the cartilage endplate for improving solute transport and disc nutrition
Source: Front Bioeng Biotechnol. 2023 Feb 27;11:1111356. doi: 10.3389/fbioe.2023.1111356 (PMC10008947; doi:10.3389/fbioe.2023.1111356)
Supplement: Supplementary file 1 [file DataSheet1.docx]

Supplementary Material

Intradiscal treatment of the cartilage endplate for improving solute transport and disc nutrition

Mohamed Habib, Shayan Hussien, Oju Jeon, Jeffrey C. Lotz, Peter I-Kung Wu, Eben Alsberg, Aaron J. Fields*

*** Correspondence:** Corresponding Author: [aaron.fields@ucsf.edu](mailto:aaron.fields@ucsf.edu)

# Cadaver MRI screening

To ensure that both motion segments from the same donor were structurally intact and of a similar degree of degeneration, we selected motion segments from levels with intact CEPs (Supplementary Figure 1) and similar Pfirrmann degeneration grades. Cadaver MRI was performed on a Discovery MR 750W 3T scanner using a Geometry Embracing Method coil contained within the table. Imaging consisted of 3D multi‐echo UTE Cones mapping sequence and a clinical T2 sequence. The multi‐echo UTE cones sequence had a repetition time of 32 ms and minimum echo time of 0.24 ms, voxel size of 0.5 x 0.5 x 3.0 mm, flip angle 17 degrees, field-of-view of 28 x 28 cm, and matrix size 560 x 560. Clinical fast spin‐echo images with T2 weighting (echo‐time 61.6 ms, repetition time 2500 ms, echo train length 8, acquisition matrix 256 × 256, slice thickness 3 mm) were also acquired in the sagittal orientation and used for Pfirrmann grading.


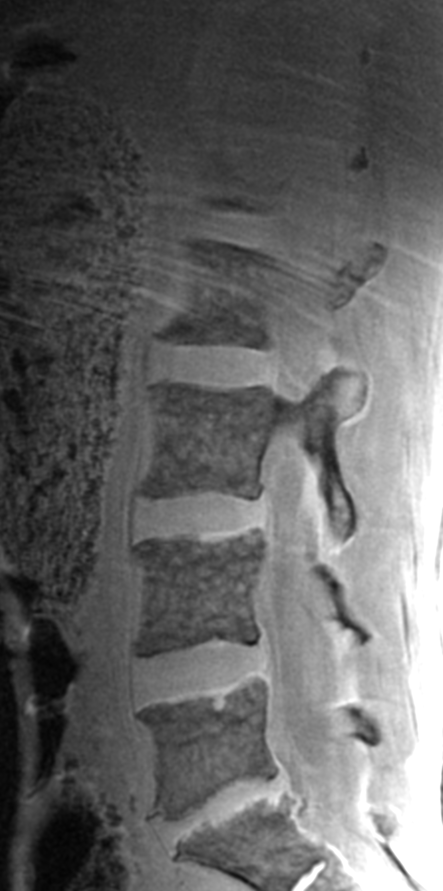


intact

damage

# Supplementary Figure 1. Sagittal UTE MR image showing discs with structurally intact and damaged CEPs

# Alginate Release Kinetics

We studied the release kinetics of a 40 kDa anionic fluorescent Dextran from three different alginate formulations; regular alginate without oxidation (0%O), alginate with 5% oxidation (5%O), and alginate with 10% oxidation (10%O). Specifically, 0.0067 mM of 40 kDa Dextran solution was prepared by dissolving 0.54 mg of 40 kDa Dextran in 2 mL of DMEM (0.27 mg/ml 40 kDa concentration). A 50 ul sample of each alginate hydrogel containing the Dextran was dispensed into 2 ml of deionized water, and the release kinetics of the of the Dextran was assessed at six time points (0.2, 1, 2, 4, 20 and 24h) with 3 experimental repeats by measuring fluorescence of the samples with a microplate reader (Ex 494 nm, Em 525 nm). Cumulative release percentage was defined as the amount of Dextran at each time point divided by the initial amount of dextran in the 50 ul sample.

We sought a hydrogel carrier that would release at least 80% of the enzyme within 4-6h of injection in order to allow enough time for the enzyme to act on the CEP during the overnight treatment period (12hr). Alginate formulations with higher oxidation levels (10%O) resulted in faster release kinetics during the initial 4-6h period than formulations with lower oxidation levels (5%O or 0%O; Supplementary Figure 2). The 40 kDa Dextran used to study the alginate release kinetics was chosen because it is in the same size range as the enzymes used in this study and in our prior study (collagenase P, 75kDa; MMP8, 52kDa), and based on the Dextran release kinetics, the 5%O and 0%O alginate formulations met our desired release criterion. In this study we used the 0%O alginate.


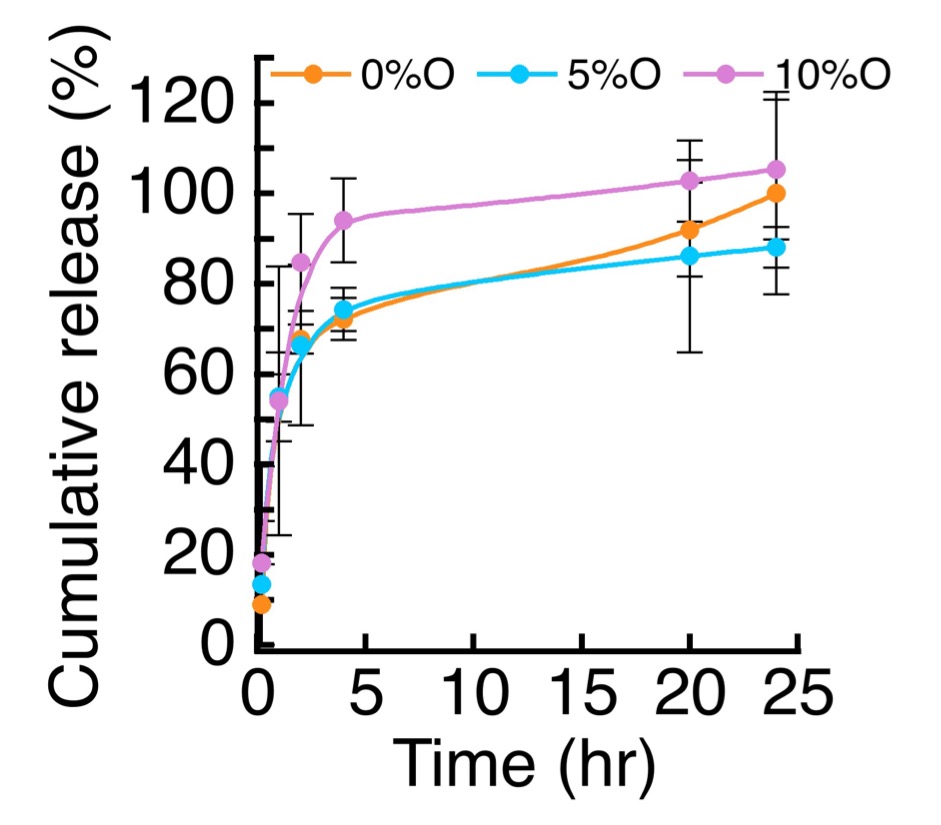


**Supplementary Figure 2.** 40 kDa dextran cumulative release (%) of alginate gels with 10%, 5% and 0% oxidation over 24h. Different alginate gels mixed with the fluorescent dextran (50 µL total volume) were placed in opaque Eppendorf tubes containing 2 mL ddH_2_O, and aliquots were assayed for fluorescence at 0.2, 1, 2, 4, 20 and 24h using a microplate reader (494 nm/524 nm ex/em).
